# Robust procedure for creating and characterizing the atomic structure of scanning tunneling microscope tips

**DOI:** 10.3762/bjnano.8.238

**Published:** 2017-11-13

**Authors:** Sumit Tewari, Koen M Bastiaans, Milan P Allan, Jan M van Ruitenbeek

**Affiliations:** 1 Huygens–Kamerlingh Onnes Laboratory, Leiden University, Niels Bohrweg 2, 2333 CA Leiden, Netherlands

**Keywords:** adatom imaging, mechanical annealing, scanning tunneling microscopy (STM), STM tip, tip apex

## Abstract

Scanning tunneling microscopes (STM) are used extensively for studying and manipulating matter at the atomic scale. In spite of the critical role of the STM tip, procedures for controlling the atomic-scale shape of STM tips have not been rigorously justified. Here, we present a method for preparing tips in situ while ensuring the crystalline structure and a reproducibly prepared tip structure up to the second atomic layer. We demonstrate a controlled evolution of such tips starting from undefined tip shapes.

## Introduction

After the advent of the scanning tunneling microscope (STM) in 1981 [1–2], it became possible to image conducting surfaces with atomic resolution. STM operates by bringing the apex of a fine metallic wire into tunneling distance from a surface of interest. By providing feedback in the tunnel current and scanning the tip over the surface one can make topographic maps of the surface with atomic resolution. STM has found its applications in many fields of science. Apart from studying surface topography, STM has been used for, e.g., manipulating single atoms [3–5], for doing spectroscopy [6], for fabricating nano-structures with novel engineered electronic properties [7], for studying the surface chemistry [8], and for probing collective [9] and local [10] electronic behavior.

It has been long known that the performance of the STM in these different fields of application is sensitive to the structure of the tip used in the measurements, since the signal is controlled by the overlap of the electronic wave functions of tip and surface. Many techniques are available for preparing atomically clean sample surfaces, including repeated cycles of sputtering and annealing in the case of metals, by fresh cleavage in the case of suitable materials, and by high-temperature annealing as for many semiconductors. However, a well defined tip structure at the atomic scale is still hard to achieve. Mechanical grinding [1], electro-polishing [11] or electrochemical etching [12–13] are standard ex situ methods for preparing microscopically sharp tips. The tip apex can be cleaned in situ using, e.g., Ar ion sputtering or electron bombardment [14], but this may disrupt the crystalline structure, which cannot be repaired by annealing since this will yield a blunt tip. For all these methods, at the atomic scale the tip structure is poorly controlled and could even have multiple local apexes. For many purposes this does not hamper STM operation, since the tunnel current decays exponentially with the tunnel gap so that the atom closest to the surface will dominate the imaging signal. However, the reproducible shape of current–voltage spectra depends strongly on the tip shape [15]. Controlled manipulation by STM of adatoms and molecules on metal surfaces depends also on the precise knowledge and reproducibility of the atomic tip structure. In the field of molecular electronics, where researchers are now trying to connect single molecules between an STM tip and a flat metal surface [16], knowledge of the tip shape is crucial.

Chen [17] has shown that the contrast of STM images depends on the choice of orbitals at the tip apex, explaining why decorating the tip apex with small molecules such as CO [18–20] has a large impact on image quality. These tip decorations [16] have been extensively studied, but very little has been done in controlling the actual tip structure itself at the atomic scale.

A first attempt in this direction was made in the pioneering article of Binning and Rohrer [1] in which they first introduced STM. They observed that the lateral resolution of their images can be increased when they gently touch the surface with the tip and then retract, which they describe as “mini-spot-welding”. Changing or preparing tips by indenting into the surface is a regular procedure used in the STM community and has been even included in some commercial STM controllers. However, the effectiveness and reproducibility of this procedure has been poorly documented and, in contrast to our method, the current during this procedure is not monitored. None of the previously reported tip forming procedures provide reproducibility, nor information about the atomic structure of the tip apex behind the apex atom.

Some studies have been carried out to determine the angular orbital symmetry [21–22] of the front atom of the tip apex by making force gradient images of a CO molecule over a Cu(111) substrate. The images are made with a tungsten tip in non-contact AFM experiments with specialized q-plus sensors. It is also shown [21] that such a combination could even yield a small asymmetry in the tunnel current in STM mode, but this asymmetry is expected to play a negligible role in tunneling between metal atoms of similar nature.

The atomic structure of STM tips can be determined ex situ in a field-ion microscope (FIM) [23–24]. However, very few studies have been made to image the tip structure in situ in STM and show its evolution upon tip preparation.

Inspiration for our current approach comes from work with low-temperature STM in cryogenic vacuum [25], where the authors report to have obtained crystalline tips by repeated deep indentation of a Au tip into a Au surface followed by retraction until the contact breaks. These indentation cycles cause plastic deformation of the tip apex [26], which first gives random conductance traces but gradually evolves to repeatable cycles, which is interpreted as evidence for a crystalline tip structure. This work on mechanical annealing was inspired by earlier break junction and STM experiments, supported by molecular dynamics simulations [25,27–28]. A first application of this approach for a Au STM tip over a graphene surface [29] was made by first locally depositing Au on the graphene surface from the tip using a high electric field pulse, followed by mechanical annealing similar to [25] over that Au deposit. The authors confirmed that the method improved the topographic contrast of the surface and the quality of the spectroscopic data.

Although this technique clearly holds promise for improvement of tip shaping and characterization, the potential has not been properly evaluated, which probably explains why it has not received more attention in the STM literature. Below we demonstrate a step by step evolution of the tip shape with a direct detection technique rather than studying conductance versus tip displacement or STM image contrast.

## Experimental

The experiments were performed in a Unisoku ultra high vacuum (UHV) and low-temperature STM with a base temperature of 2 K. The in-plane (*XY*) scan range is set by the properties of the tube piezo and is limited to 1.5 μm. The basic operations of the STM are carried out using a RHK R9 controller. This controller was coupled to a custom-written MATLAB program for the tip-annealing procedures. The sample used for the tests is a 300 nm thick Au film deposited over mica. The Au surface was prepared in situ by several cycles of argon sputtering at 1 keV at 5 · 10^−5^ mbar for 15 min and annealing at 600 K for 1 min to ensure a clean crystalline surface. The STM tips used in the experiments were commercial Unisoku platinum–iridium (PtIr) tips having a tip radius of less than 20 nm, as obtained by grinding and mechanical polishing. PtIr tips are among the most commonly used tips in STM experiments as opposed to Au tip used in reference [25]. We exploit the fact that the tips become covered with the Au sample material at the apex. We anticipate that the nanoscale tip–surface interaction will be dominated by Au at both sides, while the PtIr base provides better mechanical stability. All the measurements reported here were performed at temperatures between 2 and 4.2 K.

### Conductance traces

**Figure 1:**
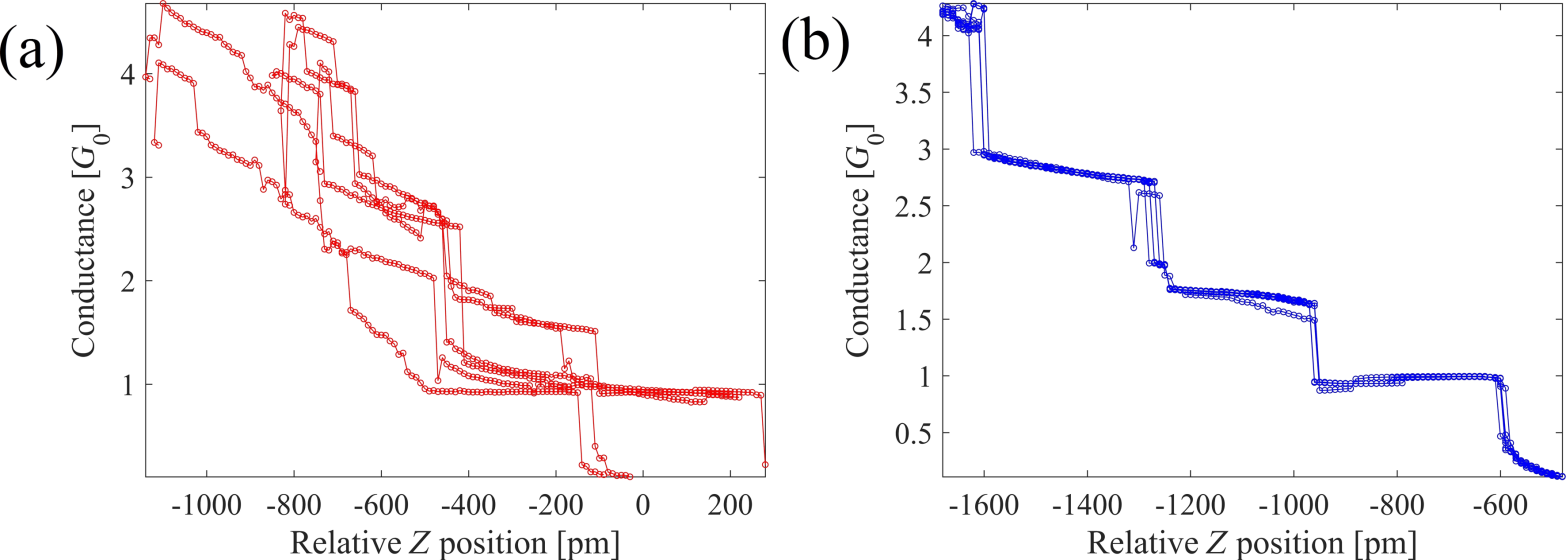
Six consecutive conductance traces of contact breaking showing (a) an initially non-repetitive structure that is converted after mechanical annealing into (b) repetitive conductance traces. The conductance is expressed in units of the quantum of conductance, *G*_0_ = 2*e*^2^/*h*.

Using this STM setup we applied training procedures for the tip apex following the findings of [25–26]. To this purpose we set up the system to complete 450 mechanical contact annealing cycles and sampled the traces in groups of 10 cycles to probe for reproducibility in the traces. Figure 1 shows examples of repetitive and non-repetitive cycles in our measurements. The system never settles permanently into either of the two cases. We see regular switching between repetitive and non-repetitive, which makes the maximum number of repetitive cycles of the order of 10–20. This appears to be different from the experiment by Sabater et al. [25] where, for a combination of Au tip and Au sample, the number of consecutively repeating traces was much larger. The lower number of repeating conductance traces may be attributed to the fact that our tip is not purely Au, but PtIr covered with Au. In the experiments of Andres et al. [29] with a graphene surface covered with a small cluster of Au, the number of repeating traces was also limited, in their case to 16. It has been reported that this repetition behavior during mechanical annealing is different for different materials [30] . Sabater [30] has studied Au,Cu, Al and Gd and found different conductance threshold values (conductance values at maximum indentation of 5*G*_0_, 6*G*_0_, 6*G*_0_ and 2.6*G*_0_, respectively) below which the conductance traces shows reproducible cycles.

### Procedure of tip preparation

**Figure 2:**
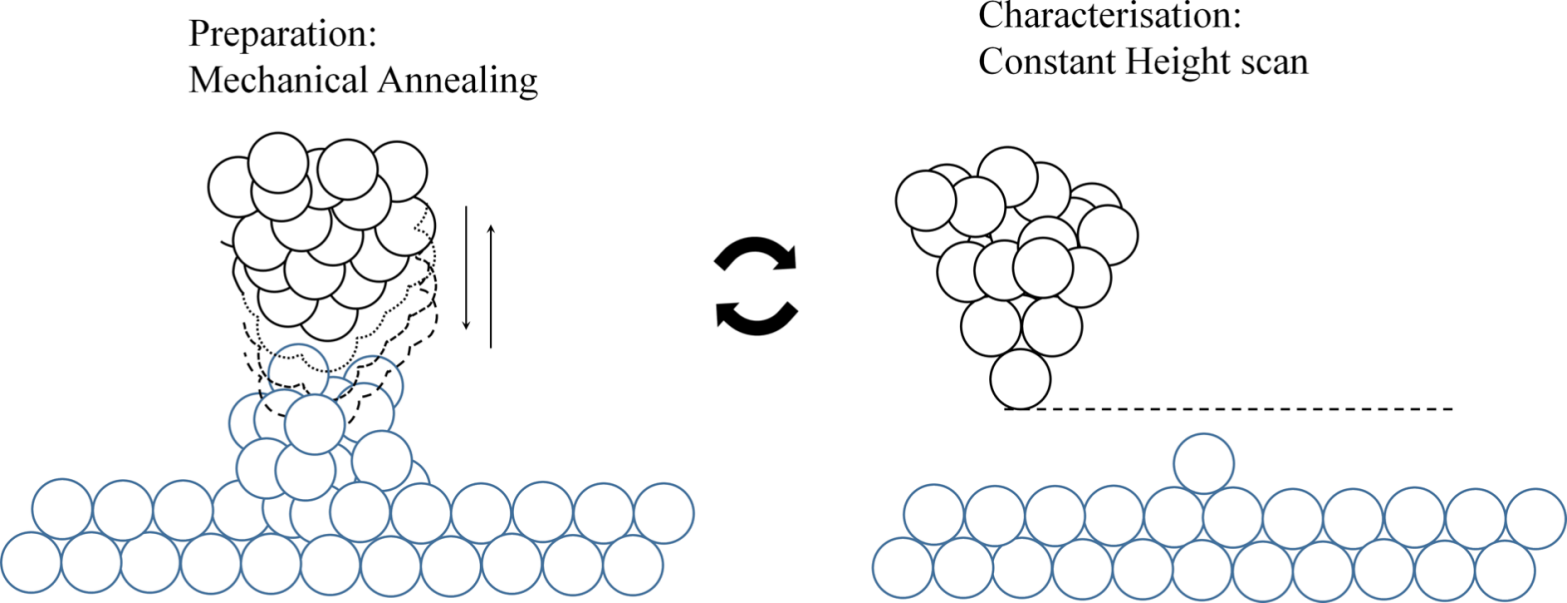
Schematic representation of the tip preparation process proposed in this article, where mechanical annealing cycles were followed by constant height scans made over a single adatom deposited on the surface.

We will now present a procedure, illustrated in Figure 2, that is based upon this mechanical annealing. This procedure permits arriving at reproducible crystalline tip shapes starting from any random initial tip shape, and we show how we can verify the evolution of the tip shape. To this end, the STM tip is indented into the flat metal surface up to a pre-set conductance value and then retracted and this cycle is repeated many times. We then exploit the capabilities of the low-temperature STM setup for imaging the evolution of the tip structure by scanning over an isolated adatom on the surface. The obtained STM images are convolutions of the topography and electronic states of sample and tip. Lang [31] has shown theoretically using two planar metal electrodes with a single adatom on each of them, that upon scanning one with the other symmetric convolutions of the topography and electronic states are expected, giving circularly symmetric images. Any asymmetry in the STM images of an isolated adatom will reflect the asymmetry of the atomic structure behind the front atom.

## Results and Discussion

**Figure 3:**
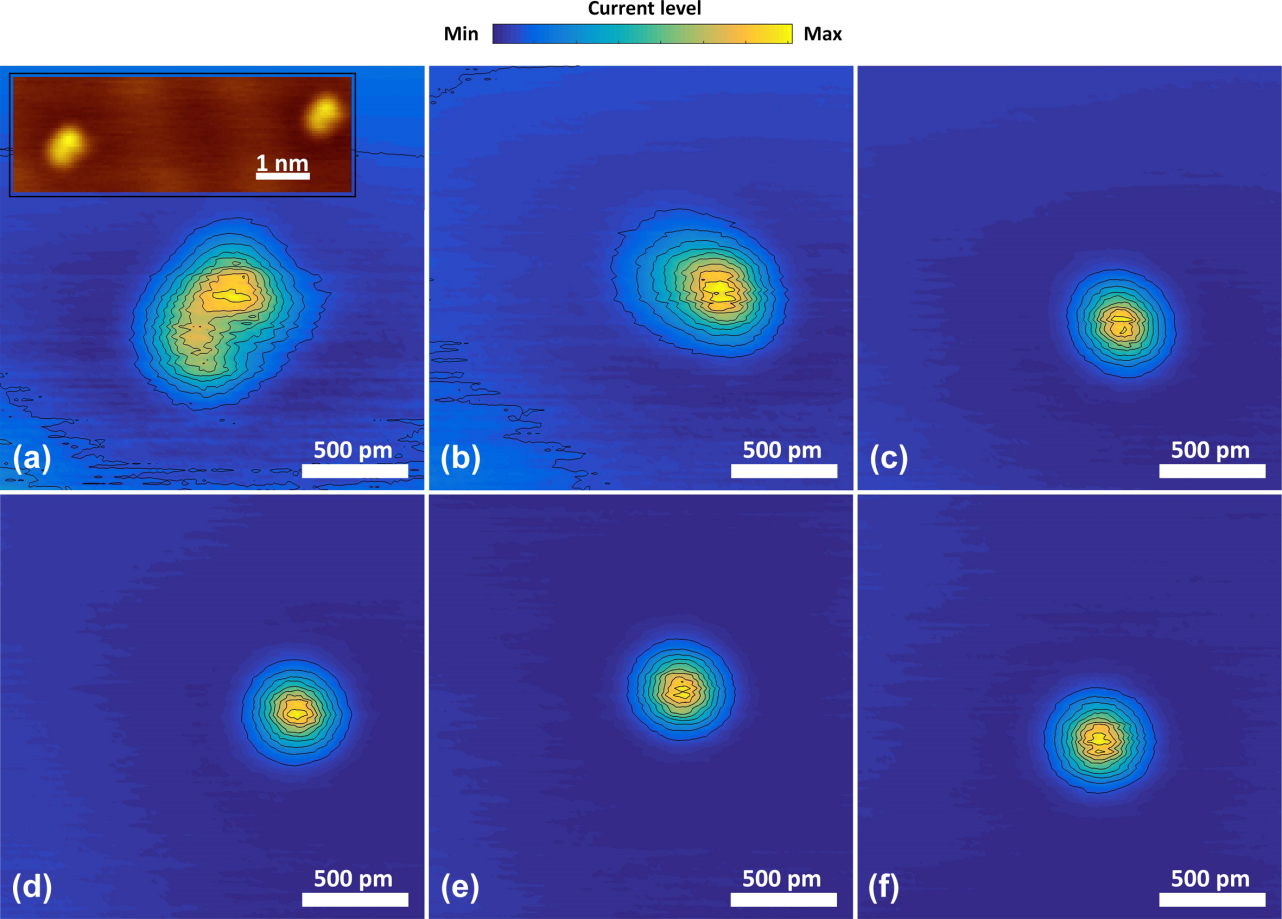
The six panels show constant-height images of a single adatom. Panel (a) shows a non-circular image of an adatom due to the random tip structure at the start. The inset of (a) shows a constant-current image of two separate adatoms prepared on the surface to confirm that the asymmetric structure is due to the tip. The evolution of tip apex is shown in the next panels, leading towards a symmetric and reproducible structure (b–f) . Between each of the images we applied 20 mechanical annealing cycles. The contours shown are linearly spaced in current. For ease of comparison the current levels in the images have been normalized to the maximum current level, which for the panels (a–f) are 66, 31, 26, 26 , 25 and 22 nA, respectively.

We start the experiments by depositing a single adatom from the Au covered PtIr tip in the center of the face-centered cubic (FCC) sector of the Au(111) herringbone reconstruction, following the procedure of [5]. At 100 mV bias, once thermal and mechanical drift have been stabilized, we release the current feedback and move the tip towards surface at a rate of 0.5 Å/s using a custom-written program in MATLAB. The motion is stopped once the conductance reaches the quantum of conductance (*G*_0_ = 2*e*^2^/*h*, which is what we expect for a single-atom point contact in Au [27]), followed by tip retraction back into the tunneling regime. This procedure leaves, with high success rate, a single Au adatom on the surface. The result is imaged in the usual topographic mode of STM shown as an inset in Figure 3a, which demonstrates that the adatom does not have circular symmetry. In order to verify that this asymmetry is associated with the tip, we deposit a second adatom. The image of the second atom is a replica of the first, in shape and orientation, confirming that the asymmetry is associated with the tip shape.

In the next steps we use an individual adatom for imaging the structure of the tip apex, employing constant-height mode scans with a box size of 2 nm centered on the adatom. The goal is to pick up the tunneling current signal from the second row of atoms above the apex atom. For FCC packing the distance to the second row is 2.5 Å larger than that to the apex atom, from which we estimate the current level to the second-row atoms at 100 pA for a current of 30 nA at the apex atom. As the tunnel current varies exponentially with distance, even small deviations from surface-parallel FCC packing of the second-layer tip atoms will give a detectable contribution. In order to scan at such high tunnel currents we first switch off the current feedback and bring the tip closer to the flat part of the surface to a fixed tunnel current value. This value is chosen to ensure that the current is as high as 30 nA, when the tip is over the adatom during the scan. Then we carry out the constant-height scan at a tip speed of 2 nm/s. Figure 3a shows an example of the resulting image at the initial stage, before starting the tip preparation procedure.

After imaging the tip apex we move the tip to an edge of the scan range, about 700 nm away from the adatom, and perform a series of 20 mechanical annealing cycles. For this we use the same MATLAB-controlled procedure as described above, except that we indent the tip farther until it reaches a preset conductance of 4*G*_0_. After 20 of these mechanical annealing cycles, we return to the original adatom and image it again by using the same procedure as above. We repeat these steps of mechanical annealing and imaging until we obtain a symmetric tip image, as illustrated in Figure 3a–f.

**Figure 4:**
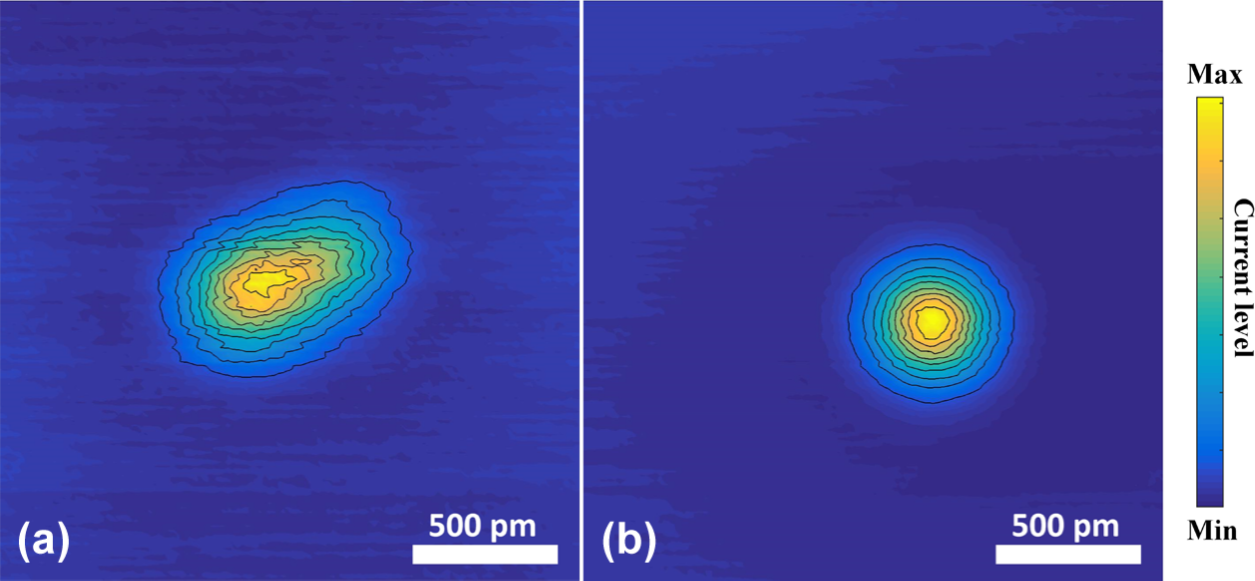
Normalized constant-height images starting from another initial tip apex (a) evolving towards a symmetric apex at the end of the procedure (b). The contours shown are linearly spaced in current. For ease of comparison the current levels in the images have been normalized to the maximum current level which for the above figures are 38 and 24 nA, respectively

The procedure for tip shaping has been applied many times in our experiments, for which the resulting tip shape was verified by the imaging of an adatom. Figure 4 shows the initial and final stage for another run. Although we occasionally observe a jump back to a more asymmetric tip configuration, the typical behavior follows a smooth evolution towards a reproducible symmetric image.

**Figure 5:**
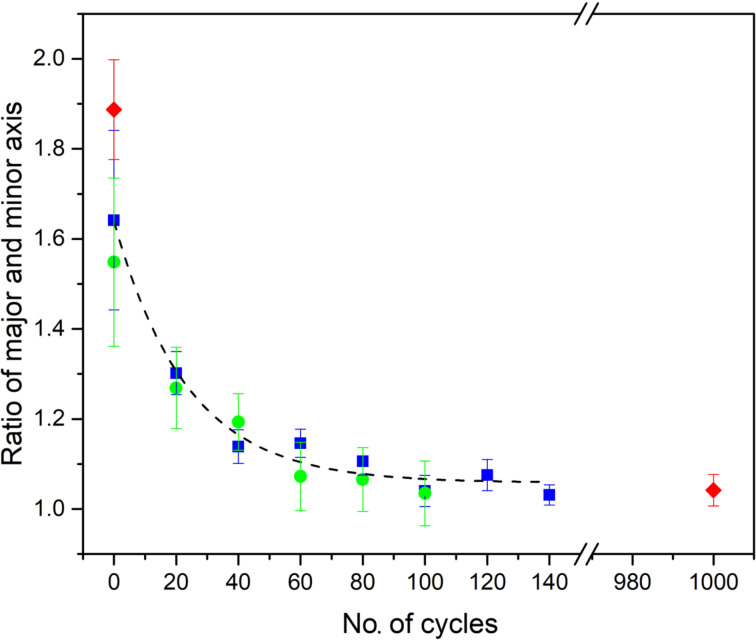
Ratio of major to minor axis of the elliptical fit showing the convergence of the tip apex structure to a circularly symmetric shape. Three independent runs are shown by blue, green and red symbols. The two points shown as red diamonds represent initial and final images of an adatom for 1000 mechanical annealing cycles. The black dashed curve is a guide to the eye showing an exponentially smooth transition to a circularly symmetric state.

For a more quantitative analysis of the results of this procedure we define a parameter that could capture the convergence of the tip structure to this symmetric state. This same parameter should also serve to verify whether tips such as those in Figure 3 and Figure 4 converged to the same state. For comparing the constant-height images we select a contour at a fixed tunnel current level (which for our case is 13.3 nA) and fit an ellipse to this contour. From the fit we extract the ratio of major to minor axis of the ellipse, *a*/*b*, as a measure of the deviation from a circular symmetry. Figure 5 shows a plot of the evolution of the ratio *a*/*b* with the number of annealing cycles. The blue and the green data points show two independent runs, for different tips and different samples. The red dots show the start and end points for a third set of data, also shown in Figure 4, applying 1000 mechanical annealing cycles. Surprisingly, the evolution of the tip is very regular and reproducible. Starting from uncontrolled and asymmetric tip apex configurations, we find that the ratio *a*/*b* decays following a common pattern, and arrives at similar minimum values. The deviation of about 4% from 1 for the value of *a*/*b* is probably limited by thermal drift and electrical noise of our system. The curve shows that the data are closely described by an exponential dependence.

## Conclusion

We have demonstrated a method for shaping a metallic tip apex in STM. By placing an adatom on a smooth Au surface the structure of the tip apex can be imaged, and we find that the shape of the STM tip evolves surprisingly smoothly and reproducibly towards an atomically sharp and symmetric structure of the second layer from the tip apex atom, starting from any random and poorly defined tip shapes. As has been illustrated by molecular dynamics simulations [25], the mechanical annealing cycles lead to a more regular atomic packing at both sides of the junction. The smooth evolution of this alignment observed here suggests that the process evolves through gradual shifts in packing and orientation of the layers farther away from the apex atom. Based on previous break junction experiments [27] it is known that for less than 100 mV bias voltage the Joule heating of the junctions is negligible and so the current level during mechanical annealing should not play an important role, when keeping the bias below this value. We find no evidence that the choice of bias voltage influences the process, although we have not tested this to much higher bias voltages. The depth of indentation is more critical: just touching the surface with the front atom is not enough, and much deeper indentation does not result in reproducible conductance cycles [30]. We have demonstrated the procedure here only for the combination of a Au-covered PtIr tip and a Au surface. However, we expect that our method is not limited to this combination of tip and sample material but is applicable to different s-wave materials, and possibly even more widely. The procedure is simple to implement in any low-temperature STM operating under UHV, and will be very useful for increasing the reproducibility of imaging and spectroscopy.

## Acknowledgements

This work was supported by the Netherlands Organization for Scientic Research (NWO/OCW), as part of the Frontiers of Nanoscience program and the Vidi talent scheme (M.P.A.).
